# New Vehiculation Systems of Mometasone Furoate for the Treatment of Inflammatory Skin Diseases

**DOI:** 10.3390/pharmaceutics14122558

**Published:** 2022-11-22

**Authors:** Raquel Taléns-Visconti, Matteo Perra, Amparo Ruiz-Saurí, Amparo Nácher

**Affiliations:** 1 Department of Pharmacy and Pharmaceutical Technology and Parasitology, Faculty of Pharmacy, University of Valencia, Av. Vicent Andrés Estellés s/n, 46100 Valencia, Spain; 2 Department of Scienze della Vita e dell’Ambiente, University of Cagliari, Via Ospedale 72, 09124 Cagliari, Italy; 3 Department of Pathology, University of Valencia, Av. Blasco Ibañez 17, 46010 Valencia, Spain; 4 Instituto Interuniversitario de Investigación de Reconocimiento Molecular y Desarrollo Tecnológico (IDM), Universitat Politècnica de València, University of Valencia, Av. Vicent Andrés Estellés s/n, 46100 Valencia, Spain

**Keywords:** mometasone furoate, glycerosomes, glyceroethosomes, glyceroethohyalurosomes, inflammatory lesions, skin diseases

## Abstract

A pre-formulation study was carried out to obtain liposomal formulations of mometasone furoate as an alternative system to marketed forms of corticosteroid for the treatment of inflammatory skin lesions. Mometasone furoate was loaded in glycerosomes and glyceroethosomes, which were also modified with hyaluronic acid (glyceroethohyalurosomes). Vesicles were designed, elaborated, and characterized, and their biocompatibility, efficacy against oxidative stress and skin lesions were assessed in vitro, in human epidermal cells, and in vivo, in a mouse skin epidermal hyperplasia model. All formulations tested showed great encapsulation efficiency, nanometric size, formed monodispersed systems and a highly negative Z potential. Similar values were obtained over nine months storage at 4 °C, which indicates the great stability of the three types of nanoliposomes at least during the time tested. Among them, 0.1% mometasone furoate glyceroethohyalurosomes were the best formulation to protect cells against oxidative stress and their anti-inflammatory efficacy was confirmed in vivo, being even more effective than the marketed form (Elocom^®^), as the reduction in the inflammation was even ~15% higher than that achieved with the commercial cream. Selected formulations could be potential candidates as new vehiculation systems for mometasone furoate. The presence of hyaluronic acid in glyceroethohyalurosomes makes them the best candidates in preventing/treating skin inflammatory lesions.

## 1. Introduction

Inflammatory diseases are among today’s major medical problems, as many of them are caused by an uncontrolled and continuous activation of inflammatory responses that cause damages to organs and tissues. Thus, nowadays, among the main causes of medical consultation are pain and inflammation associated with a wide range of disorders. These disorders include psoriasis, a chronic, inflammatory, immune-mediated, and relapsing disease of unknown etiology [1,2]. In fact, psoriasis is now considered a systemic disorder with predominantly cutaneous manifestations. It has peculiarities such as its relapsing–remitting course, diversity of clinical presentations and severity [3]. Its incidence increases with age, showing two peaks of incidence, in young–middle age and in late life [4]. Clinically, psoriasis usually presents with very dry, hyperkeratotic plaques in various locations, including the scalp, which can interfere with work and personal life, as the skin is often cracked, bleeding and painful [5]. Moreover, cases of new-onset or exacerbation of psoriasis were recently reported as cutaneous adverse events following COVID-19 vaccination [6].

Psoriasis is characterized by a high prevalence, chronicity, disfigurement, disability and co-morbidity [1]. In fact, the negative impact on the quality of life of psoriatic patients is considered comparable to that caused by ischemic heart disease, diabetes, depression, or cancer [7,8]. It is estimated to affect approximately 2–4% of the world’s population. The high prevalence of psoriasis is an important consideration for the WHO. Thus, at the 67th World Health Assembly, through a resolution adopted, its Executive Committee highlighted psoriasis as a crucial health problem. It also urged Member States to strengthen public awareness of this disease and to fight the stigmatization of people afflicted by it [9,10]. The resolution also called for increased research and identification of strategies to integrate the treatment of psoriasis [5,11].

Different topical and systemic treatments are available for psoriasis, with drugs from various pharmacological groups. Their choice depends on several factors, such as severity, extent of the lesion and response to therapy. Topical treatments are usually the first choice and only in the most severe cases are they combined with systemic and/or biological treatments, as, for example, in the case of extensive psoriasis that does not respond to other treatments (erythrodermic and pustular forms and disabling forms). For severe cases, cyclosporins, methotrexate, hydroxyurea, retinoids and biological immunomodulators are mainly administered [4,12,13]. Although the use of biological treatments has increased in recent years, they are reserved for the most complex cases, as they represent a high percentage of the hospital budget.

Topical therapies are the cornerstone in the treatment of mild to moderate psoriasis or with limited extension. Topical corticosteroids represent the first-line option due to their greater efficacy. They are characterized by high anti-inflammatory and immunosuppressive activity, clearing and reducing plaques. Regarding their use, the greatest risk lies with high-potency corticosteroids, in particular when they are administered over large skin areas, for a prolonged period of time or under occlusion due to the local and systemic adverse effects, if they enter systemic circulation (an increase in blood glucose levels, an increase in blood pressure, a decrease in blood potassium levels, osteoporosis, etc.), which may develop. Consequently, they should be used for short periods of time or, in the case of prolonged treatments, at very low doses, which can lead to treatment abandonment or poor compliance and therefore to treatment failure. Alternatively, corticosteroids can be replaced by retinoids or vitamin D analogues, even though they are quite irritating to damaged skin [14,15]. Among the goals of this work is to contribute to overcoming these limitations and to establish possible therapeutic alternatives.

In this context, the development of new topical delivery forms that contribute to increasing the efficacy and safety of conventional drugs is justified. Currently, nanoformulations, modified-release forms, represent a promising approach for the safe and effective treatment of psoriasis [16,17,18,19]. The selection of the proper vehicle plays an important role as it can contribute to improve skin hydration and repair, as well as the penetration of the drugs into the skin, which determines their efficacy. In the treatment of psoriasis, the use of emollients and moisturizers is recommended, as they are necessary to prevent the dryness and flaking of the skin associated with the disease [20,21,22,23]. In this regard, nanovesicles coated with biopolymers (such as hyalurosomes) can contribute significantly to therapeutic innovation in psoriasis [17,24]. Some of the excipients that constitute liposomes have these actions. On the other hand, lotions and gels are the most commonly used vehicles in the treatment of localized lesions on the scalp, although they can be irritating and excessively drying to the skin. Replacing these vehicles with liposomes could help to avoid or reduce these phenomena.

**Figure 1:**
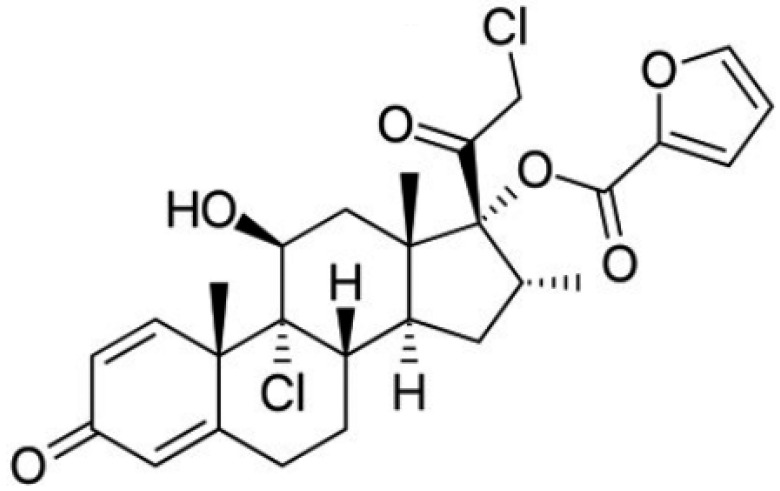
Structure of Mometasone furoate [29].

Among the corticosteroids, mometasone furoate (MF), with the structure shown in Figure 1, should be highlighted. It is an important topical corticosteroid that is used in the treatment of several skin disorders such as dermatitis or psoriasis. Topical corticosteroids tend to reduce the thickness of the skin which turns favorable in the case of psoriasis [25,26]. In this sense, MF has proved to be effective against psoriasis vulgaris in a once-a-day topical application [27]. In fact, MF has high potency and low risk of developing local and systemic adverse effects. Further, its anti-inflammatory activity and duration are superior to that of betamethasone [28]. Clinical trials have shown that mometasone furoate has comparable or significantly better efficacy in all indications studied in both adults and children. It is well tolerated, with mild to moderate transient local adverse effects. It is characterized by low systemic bioavailability, due to its high lipophilicity, low percutaneous absorption and rapid hepatic biotransformation, so it does not have a significant effect on the hypothalamic–pituitary–adrenal axis. The cutaneous biotransformation of mometasone furoate results in a lower affinity for dermal cells than epidermal cells, which contributes to its low anthropogenicity. In addition, sensitization to mometasone furoate is low [29].

The main objective of this study was to develop new vehiculation systems of mometasone furoate for the treatment of inflammatory skin diseases. To achieve this, different mometasone furoate nanovesicles have been designed, developed, and characterized. Selected formulations were further analyzed for stability studies. Moreover, in vitro biocompatibility and protective effect against oxidative damage of the proposed formulations were determined. In addition, the in vivo efficacy was assessed in a mouse skin epidermal hyperplasia model.

## 2. Materials and Methods

### 2.1. Materials

Lipoid S75 (S75), a mixture of soybean phospholipids (~70% phosphatidylcholine, 9% phosphatidylethanolamine and 3% lysophosphatidylcholine), triglycerides and fatty acids, was purchased from Lipoid GmbH (Ludwigshafen, Germany). Mometasone furoate (Carbosynth^®^), mometasone furoate commercial cream (1 mg/g Elocom^®^ cream), ethanol (Guinama^®^), glycerol (Acofarma^®^), Tween 80 (Scharlab S.L., Barcelona, Spain), vitamin E (Guinama^®^), PBS (VWR Life Science^®^) and hyaluronic acid (Carbosynth Limited (Compton, UK)), phorbol 1,2-myristate 1,3-acetate (Sigma-Aldrich, St. Louis, MI, USA), and all solvents of analytical grade were purchased from (Scharlab S.L). Cell medium, fetal bovine serum, penicillin, and streptomycin, and all other reagents for cell studies, were purchased from Thermo Fisher Scientific Inc. (Waltham, MA, USA).

### 2.2. Vesicle Preparation

In Table 1, vesicle composition is reported. Briefly, S75 and mometasone furoate (1 mg/mL) were weighed in a glass vial, and Tween 80 and vitamin E were added. These were hydrated with glycerol, ethanol, and PBS or 0.1% hyaluronic acid in PBS. Dispersions were left hydrating overnight, to promote the swelling of the phospholipid, and then left into an ultrasound bath, for 5 min at 35 °C. After that, dispersions in well-sealed vials to avoid ethanol evaporation, were left in a water bath at 60 °C for 10 min, to overcome the transition temperature of the phospholipid. The dispersions were sonicated (5 + 5 + 3 min cycles, 5 s on and 2 s off, with a probe amplitude of 64%, 44% and 64%, respectively, allowing the sample cooling between each sonication), by using an Optic ivymen^®^ System ultrasonic disintegrator, to obtain homogeneous systems with small particles. At the end of the sonication process, liposomes were extruded with an Avanti^®^ Mini-Extruder (Avanti Polar Lipids, Alabaster, Alabama) through a 200 nm membrane (Whatman, GE Healthcare, Fairfield, CO, USA) to obtain an estimated final diameter of 200 nm. Empty formulations were also prepared and used as references.

### 2.3. Vesicle Characterization

The average diameter and polydispersity index of the vesicles were determined in triplicate by Photon Correlation Spectroscopy using a Zetasizer Nano-S^®^ (Malvern Instruments, Worcester-shire, UK) at 25 °C. The Zetasizer was also used to measure the surface charge of vesicles (zeta potential) measuring their electrophoretic mobility in dispersion by electrophoretic light scattering in a thermostated cell. Each sample was diluted (1:30) to be optically clear and to avoid the attenuation of the laser beam by the particles along with the reduction in scattered light that can be detected.

The entrapment efficiency was calculated by measuring mometasone furoate concentration into vesicles before and after their purification from the unentrapped drug by dialysis. To achieve this, vesicle dispersions (1 mL) were loaded into dialysis tube (Spectra/Por^®^ membranes, 12–14 kDa MW cut-off, 3 nm pore size; Spectrum Laboratories Inc., DG Breda, The Netherlands) and maintained at room temperature in one liter of water for 2 h, refreshing water after 1 h. The mometasone furoate concentration of dispersions before and after dialysis was measured. For this, both non-dialyzed and dialyzed samples were treated with methanol (1:100) and the MF content was analyzed by a high-performance liquid chromatography (HPLC) Perkin Elmer^®^ Series 200 equipped with a photodiode array UV detector. 20 µL of the samples were injected in a C18 reverse-phase column (Teknokroma^®^ Brisa “LC2” 5.0 µm, 150 mm × 4.6 mm). The isocratic mobile phase was a mixture of 1% glacial acetic acid solution and acetonitrile (10:90, *v*/*v*) and the flow rate 1 mL/min. The detection wavelength was set at 254 nm. Standard calibration curves covering the whole MF range concentrations in experimental samples were obtained. The limit of detection and quantification was 0.22 µg/mL and 0.65 µg/mL, respectively.

The drug entrapment efficiency (*EE*%) was calculated as follows (Equation (1)):

(1) $$EE\;\left(\%\right)=\left(\frac{actual\;drug}{initial\;drug}\right)\times 100$$

where *actual drug* is the amount of MF in vesicles after dialysis, and *initial drug* is the amount of MF before dialysis, as calculated by HPLC.

Finally, the stability of the vesicles was assessed by monitoring their mean diameter, polydispersity index and Z potential over 9 months at 4 ± 1 °C.

### 2.4. In Vitro Cytotoxicity of Formulations

Human epidermal cells keratinocytes (HaCaT, ATCC collection, Manassas, VA, USA) were grown as monolayers in 150 cm^2^ flasks, incubated with 100% humidity and 5% CO_2_ at 37 °C. Phenol red-free Dulbecco’s Modified Eagle Medium (DMEM) with high glucose, supplemented with 10% fetal bovine serum and penicillin/streptomycin, was used to culture keratinocytes. The cells were seeded into 96-well plates at a density of 5 × 10^4^ cells/well and after 24 h of incubation, were exposed for 48 h to the mometasone furoate loaded vesicles properly diluted to reach different concentrations (1, 0.1, 0.01, 0.001 µg/mL). MF dispersion at the same dilutions was used as reference. At the end of the experiments, the cells were washed with warmed phosphate buffer solution and their activity was measured using the MTT [3(4,5-dimethylthiazolyl-2)-2, 5-diphenyltetrazolium bromide] colorimetric assay. MTT solution (100 µL 0.5 mg/mL in PBS, final concentration) was added to each well, and cells were incubated for 3 h. Af-ter that, the formed formazan crystals were dissolved in 100 µL of dimethyl sulfoxide and their concentration was spectrophotometrically quantified at 570 nm by using a microplate reader (Multiskan EX, Thermo Fisher Scientific, Inc., Waltham, MA, USA). All experiments were repeated at least in triplicate and results are shown as percent of cell viability in comparison with non-treated control cells (100% viability) (Equation (2)):

(2) $$Cell\;Viability\;\left(\%\right)=\left(\frac{Absorbance_{sample}}{Absorbance_{control}}\right)\times 100$$

where *Absorbance_sample_* is the absorbance of the correspondent cells treated with the correspondent formulation and *Absorbance_control_* is the absorbance of non-treated cells.

### 2.5. In Vitro Protective Effect of Formulations against Oxidative Damage in Keratinocytes

The ability of the formulations to protect keratinocytes from damages induced by hydrogen peroxide was evaluated. Cells were seeded in 96-well plates (5 × 10^4^ cells/well) and incubated at 37 °C in 5% CO_2_ for 24 h. Cells were stressed with hydrogen peroxide (30% diluted 1:50,000 *v/v* with medium) and treated for 4 h with the MF in dispersion or loaded in vesicles opportunely diluted to reach 1, 0.1, 0.01, and 0.001 µg/mL of drug. Cells stressed with hydrogen peroxide and untreated were used as the negative control, health cells unstressed and untreated were used as the positive control. At the end, the cells were washed with phosphate buffer solution and the MTT assay was used to assess the viability as described previously.

### 2.6. Evaluation of Protective Effect of Mometasone Furoate Vesicles against Skin Damage In Vivo

Animals selected for the experiments were CD-1 female mice, 5–6 weeks old and 25–35 g of weight supplied by Envigo laboratories (Barcelona, Spain). Before performing the experiments, mice were acclimatized for ∼7 days. All the experiments were carried out according to the European regulations concerning the handling and use of experimental animals, and the protocols were approved by the Institutional Animal Care and Use Committee of the University of Valencia (2021/VSC/PEA/0178). CD-1 female mice (*n* = 4) were divided in different groups: untreated animals as the negative control, animals treated with 12-O-tetradecanoylphorbol 13-acetate (TPA) and saline solution as the positive control, and animals treated with both TPA and the formulations or a commercial mometasone furoate cream (1 mg/g Elocom^®^ cream). One day before the experiments, the back skin of mice was shaved, obtaining a shaved area of ∼2 cm^2^. During the first day, skin damages were induced by applying 20 μL of TPA dissolved in acetone (243 μM) on the shaved area. After 3 h, samples (200 μL) were gently applied over the TPA-treated area allowing the complete absorption. The procedure was repeated for additional 2 days. Mice were sacrificed by cervical dislocation, and samples of the treated skin area were excised and immediately stored at −80 °C.

To determine the neutrophil infiltration into the skin, myeloperoxidase (MPO) activity was measured as previously described [30]. Skin samples were homogenized and centrifuged, the supernatant was diluted 1:10 with sodium phosphate buffer (pH 5.4). Then, 10 µL of the diluted supernatant was incubated with 20 µL of sodium phosphate buffer (pH 5.4), 200 µL of phosphate buffer (pH 7.4), 40 µL of 0.052% hydrogen peroxide and 20 µL of 3,30,5,50-tetramethylbenzidine dihydrochloride (18 mM). To stop the reaction, 50 µL of H_2_SO_4_ (2 N) was added. MPO activity, expressed as ng/mL, was determined from the linear portion of a standard curve by reading the absorbance at 450 nm.

### 2.7. Histological Examination

Skin biopsies (see paragraph 2.6) were excised from the treated mice dorsal region, after 72 h of treatment (on day 4) and maintained in formaldehyde (10% *v*/*v*) for microscopic studies. Tissue samples were processed routinely and embedded in paraffin wax. Longitudinal sections (5 μm) were stained with hematoxylin and eosin. Microscopic assessment by light microscope was performed blind on coded slices.

### 2.8. Statistical Analysis of Data

Results are expressed as the mean ± standard deviation. Analysis of variance (ANOVA) was used for multiple comparisons of means, and Tukey’s test and Student’s *t*-test were performed to substantiate differences between groups using IBM SPSS Statistics 28.0.1.1 for Windows. The differences were considered statistically significant for *p* < 0.05.

## 3. Results and Discussion

### 3.1. Vesicle Preparation and Characterization

A pre-formulation study was carried out using different amounts of phospholipid, tensioactive and water cosolvents (Table 1). Given their composition, vesicles were named glycerosomes, glyceroethosomes and glyceroethohyalurosomes. Glycerosomes were formulated because they can effectively improve the stability of traditional phospholipid vesicles, by adding glycerol, due to their higher viscosity [31,32]. Glyceroethosomes, also known as glycethosomes, emerge as a way to combine the advantages of glycerosomes and ethosomes, that by adding ethanol as a penetration enhancer can promote drug distribution in the skin as it fluidizes the phospholipid bilayers [33,34,35]. Moreover, alcohols may also improve the solubility of drugs [36]. In this work, both glycerol and ethanol were used for all these purposes. Furthermore, glyceroethosomes were modified by the addition of hyaluronic acid (glyceroethohyalurosomes), which is expected to ameliorate the adhesion of the vesicles to the skin and aid wound restoring [37]. Thus, glyceroethohyalurosomes were formulated as an innovative tool for preventing and treating skin affections.

Therefore, a total of six formulations were designed and characterized in terms of encapsulation efficiency, size, polydispersity index and Z potential. Results are shown as the mean and standard deviation of six replicates for each condition (Table 2). Among the six formulations tested, vesicles were of nanometric size (<180 nm), the polydispersity index was ≤0.25, confirming the formation of monodispersed systems, and the Z potential was always highly negative (≤−25 mV), which indicates that a difficulty in the subsequent aggregation of the particles is to be expected, helping to maintain the stability of the system. Nevertheless, only the vesicles made of Lipoid S75 (12%), MF (1 mg/mL) and Tween 80 (0.24%) were able to load the active in high quantities, as indicated by the entrapment efficiency values (>82%), with significant differences when compared with the corresponding formula with higher proportion of Lipoid S75 (18%) and Tween 80 (0.57%), *p* < 0.01 in all cases. For this reason, formulations F1, F2 and F3 were selected for further studies. Additionally, two more batches were prepared and subjected to the same characterization tests. No significant differences were observed between batches.

**Figure 2:**
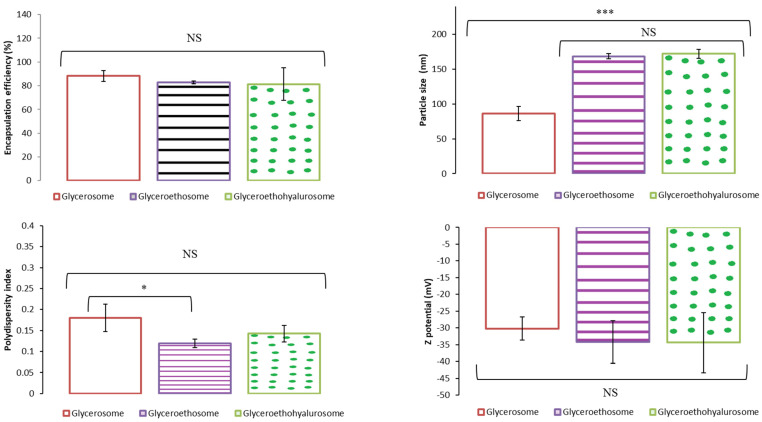
Mean encapsulation efficiency, particle size, polydispersity index and Z potential of selected mometasone furoate-loaded vesicles. Data are represented as the mean values ± standard deviations (*n* = 3). (NS no significative differences; * *p* < 0.05; *** *p* < 0.001.)

Figure 2 shows the encapsulation efficiency, size, polydispersity index and Z potential results obtained as the mean and standard deviation of three replicates of the three batches tested for glycerosomes, glyceroethosomes and glyceroethohyalurosomes. As can be observed, the percentage of mometasone furoate-loaded in each of the types of nanovesicles was between 88.19% and 81.33%, with no statistically significant differences among them. Moreover, glycerosomes were the system with the smallest size compared to glyceroethosomes and glyceroethohyalurosomes (*p* < 0.001), although in all cases nanoliposomes were obtained. In terms of polydispersity index, and despite the statistically significant differences observed among nanovesicles, the value is always lower than 0.2, showing the high homogeneity of the systems. Finally, the Z potential of the three types of formulation does not show significant differences among them (*p* > 0.05) and in all cases were highly negative.

**Figure 3:**
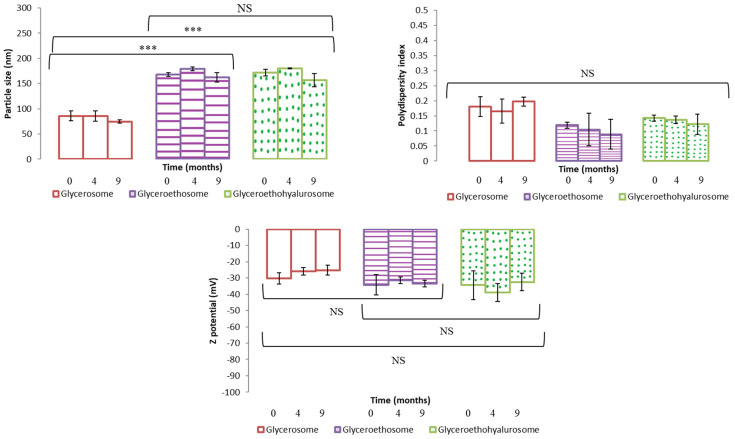
Mean particle size, polydispersity index and Z potential of selected mometasone furoate-loaded vesicles measured during 9 months of storage at 4 °C. Data are represented as the mean values ± standard deviations (*n* = 3). (NS no significative differences; *** *p* < 0.001.)

The obtained dispersions were stored at 4 °C, in dark for 9 months. To evaluate nanovesicles stability over time, their mean diameter, polydispersity index and Z potential were measured at scheduled times. Results are shown as the mean and standard deviation of the three batches in Figure 3. As can be noticed, this study showed a good stability in the three types of nanovesicles. In fact, the tested parameters remained almost unchanged over 9 months of storage for all the formulations. These results are in good agreement with those found in the literature for similar vesicles [36,38].

### 3.2. Biocompatibility of Formulations

The in vitro biocompatibility of vesicular systems has to be proven before the evaluation of efficacy to confirm their effective safety. In this study, the evaluation of the biocompatibility of the formulations was performed in vitro by using keratinocytes (HaCaT). The viability of cells was measured by performing the MTT assay, considering untreated cells as control.

**Figure 4:**
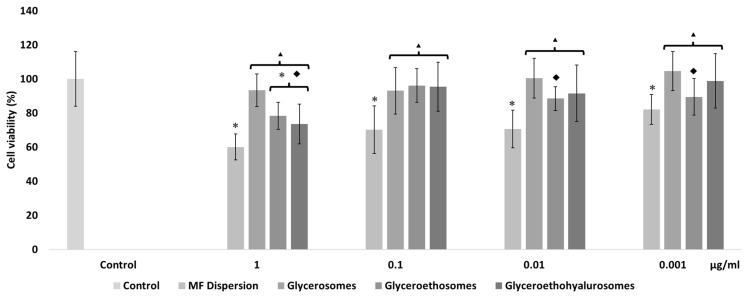
Cell viability of keratinocytes treated for 48 h with mometasone furoate in dispersion or loaded in vesicles diluted to reach 1, 0.1, 0.01, and 0.001 μg/mL of drug. Data are reported as the mean values ± standard deviations of cell viability expressed as the percentage of untreated cells (100% of viability). The symbol * indicates values that were statistically different from control; the symbol ▴ indicates values that were statistically different from mometasone furoate dispersion; the symbol ◆ indicates values that were statistically different from glycerosomes (*p*  <  0.05).

The mometasone furoate-loaded vesicles were appropriately diluted with the cell medium and the MF in dispersion at the same dilution was used as reference aiming at understanding the effect of both MF and carriers on cell viability (Figure 4). The viability of HaCaT cells treated with the MF dispersion at the highest concentration was ~60%, and slightly increased up to ~70% with MF at 0.1 and 0.01 µg/mL. At the lowest concentration of the dispersion, cell viability was ~80%. The viability of cells treated with glycerosomes was ~100%, irrespective of the used concentration. At the highest concentration, the viability of cells treated with glyceroethosomes and glyceroethohyalurosomes was ~80% and ~70%, respectively. HaCaT viability treated with lower concentrations of glyceroethosomes and glyceroethohyalurosomes, was ~90% and ~100%, respectively%.

In summary, although glycerosomes showed the highest biocompatibility in the different concentrations used, as previously demonstrated by Manca et al. [39], all three types of vesicles showed significant differences in cell viability when compared with MF dispersion, confirming the high biocompatibility of the proposed formulations. In fact, all liposomal forms protected the cytotoxic effect of MF in dispersion which makes them as promising dermal delivery system.

### 3.3. Protective Effect of the Formulations against Damages Induced by Hydrogen Peroxide

The exposition of cells to hydrogen peroxide (H_2_O_2_) causes cell apoptosis and death, due to the production of ROS [40].

**Figure 5:**
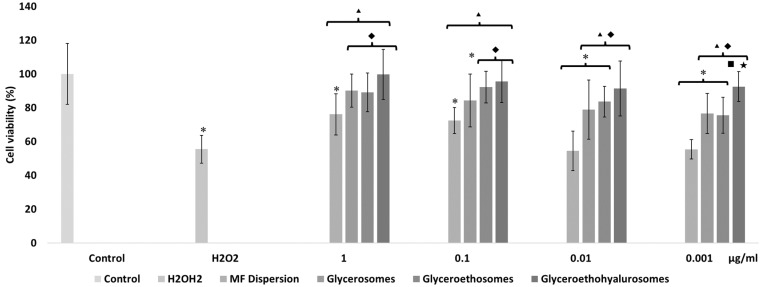
Viability of keratinocytes stressed with hydrogen peroxide and protected with mometasone furoate in dispersion or loaded in vesicles properly diluted to reach 1, 0.1, 0.01, and 0.001 μg/mL of drug. Data are reported as the mean values ± standard deviations of cell viability expressed as the percentage of untreated cells (100% viability). The symbol * indicates values that were statistically different from control; the symbol ▴ indicates values that were statistically different from hydrogen peroxide; the symbol ◆ indicates values that were statistically different from mometasone furoate dispersion; the symbol ■ indicates values that were statistically different from glycerosomes; the symbol ★ indicates values that were statistically different from glyceroethosomes (*p*  <  0.05).

The in vitro treatment of cells with H_2_O_2_ is a reliable method to assess the ability of drugs to prevent or inhibit the development of oxidative damages. According to this, keratinocytes were stressed with hydrogen peroxide and simultaneously protected with mometasone furoate free or loaded in vesicles (Figure 5).

The activity of cells stressed with hydrogen peroxide decreased up to ~55%, that of cells stressed and simultaneously treated with the drug dispersion was the same (~55%) using lower concentrations of MF (0.01 and 0.001 mg/mL) and become higher using 1 and 0.1 µg/mL of drug, respectively ~76% and ~72%. This indicates that at higher dilutions, the free drug was not able to protect the keratinocytes against the reactive species generated by hydrogen peroxide. Furthermore, at lower dilutions, the protective effect of mometasone furoate dispersion was mild. When treating stressed cells with the lowest concentration of glycerosomes and glyceroethosomes (0.001 µg/mL), cell viability was ~76%, demonstrating the protective effect of these vesicles. Indeed, MF ability to inhibit the formation and propagation of free radicals was enhanced by its loading into glycerosomes and glyceroethosomes vesicles at the highest concentration (1 µg/mL), as the cell viability was higher than ~90%. The loading of MF in glyceroethohyalurosomes provided the highest protection against oxidative damages, since cell viability was higher than ~90%, irrespective of the used dilution. In particular, at the lowest dilution of glyceroethohyalurosomes were able to restore H_2_O_2_ stressed cells viability up to 100%.

### 3.4. Evaluation of Protective Effect of Mometasone Furoate Vesicles against Skin Damage In Vivo

**Figure 6:**
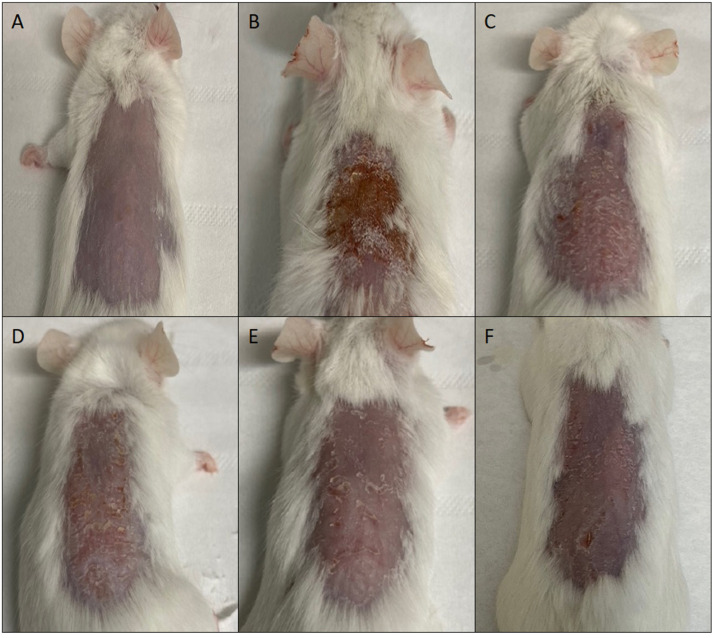
Representative images of mouse skin: untreated (**A**), damaged by TPA and treated with saline solution (**B**) or treated with mometasone furoate commercial cream (**C**), or mometasone furoate loaded in glycerosomes (**D**), glyceroethosomes (**E**) and glyceroethohyalurosomes (**F**).

Mometasone furoate-loaded vesicles were tested in a mouse skin epidermal hyperplasia model using a commercial cream (Elocom^®^ cream) that was used as reference. The skin of mice injured by TPA and treated with saline solution (positive control), appeared desquamated and covered with necrotic tissue (Figure 6).

The skin of mice treated with MF commercial cream, or MF loaded in glycerosomes and glyceroethosomes was almost healed, but still showing some desquamation and minor lesions. On the other hand, glyceroethohyalurosomes appeared to be the most effective formulation, as the skin appeared to be practically healed by TPA-induced damage.

**Figure 7:**
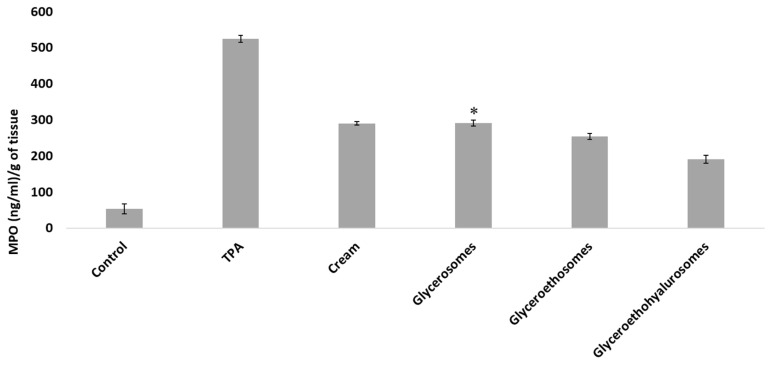
Myeloperoxidase (MPO) concentration in mice untreated (Control), exposed to TPA and treated with saline (TPA) or treated with mometasone furoate commercial cream (Cream), or mometasone furoate loaded in glycerosomes (D), glyceroethosomes (E) and glyceroethohyalurosomes (F). Mean values ± standard deviations are reported. The symbol * indicates same values as mometasone furoate commercial cream (*p* > 0.05).

Myeloperoxidase activity was measured to evaluate the entity of inflammation at skin level. Results are shown in Figure 7. The MPO concentration in the untreated tissue was ~50 (ng/mL)/g_of tissue_, that of inflamed by TPA application and treated with saline solution was ~520 (ng/mL)/g_of tissue_. The treatment with the same MF dose incorporated in a commercial cream, glycerosomes and glyceroethosomes determined a decrease in the skin inflammation, as the MPO concentration were ~290 (ng/mL)/g_of tissue_ for the cream and glycerosomes and ~250 (ng/mL)/g_of tissue_ for glyceroethosomes. The most significant reduction in myeloperoxidase was observed when mice were treated at the same conditions and MF dose with glyceroethohyalurosomes, as MPO concentration was ~190 (ng/mL)/g_of tissue_. A possible explanation for this observation could be that hyaluronic acid may improve the vesicle bioadhesiveness as well as act as a targeting moiety to CD 44 epithelial cells, overexpressed in inflammatory tissues [41,42].

### 3.5. Histological Examination

**Figure 8:**
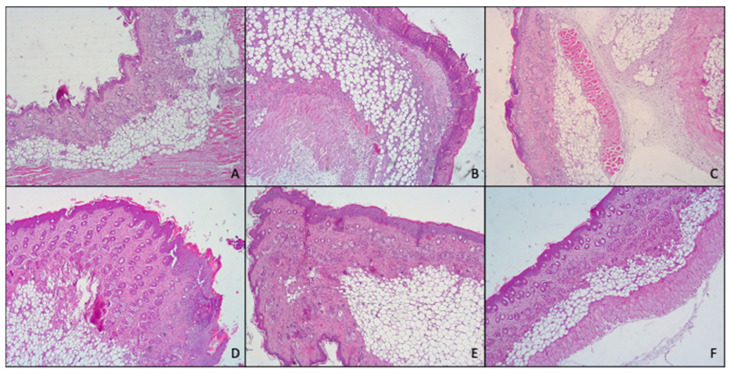
Representative images of the histological determination of mouse skin: untreated (**A**), damaged by TPA and treated with saline solution (**B**) or treated with mometasone furoate commercial cream (**C**), furoate loaded in glycerosomes (**D**), glyceroethosomes (**E**) and glyceroethohyalurosomes (**F**).

Histological analysis (Figure 8) confirmed what previously found with both macroscopic observation of mice skin and myeloperoxidase activity.

In fact, skin treated with TPA, displayed the presence of numerous important pustules with involvement of the entire epidermis and severe exocytosis. Furthermore, epidermal necrosis and the presence of edematous fluid in the dermal–epidermal junction due to the separation of both layers were noted. Hyperkeratosis and severe inflammatory infiltrates in the dermis that reached the hypodermis, and the muscle were also observed.

However, topical application of MF-loaded glycerosomes and glyceroethosomes were able to effectively reduce the damages induced by TPA, exerting similar results to those of the commercial cream, as all of them showed hyperkeratosis, slight or moderate inflammatory infiltration in dermis and panniculitis and exocytosis.

Further, when MF was loaded in glyceroethohyalurosomes, the protection against inflammatory damages induced by TPA increased, as only a moderate hyperkeratosis, inflammatory infiltration and slight panniculitis were detected.

In summary, when considering together in vivo results (MPO analysis and histological examination), 0.1% mometasone furoate glyceroethohyalurosomes are the best candidate in preventing/treating skin inflammatory lesions. This assertion is based on the observation that, although skin treatment with MF cream, glycerosomes and glyceroethosomes decrease MPO levels ~50% compared to skin treated with TPA, macroscopic observation still showed hyperkeratosis, slight or moderate inflammatory infiltration in dermis and panniculitis and exocytosis. However, when MF was loaded in glyceroethohyalurosomes the highest reduction, ~65%, in MPO levels was found. This finding is in line with those observed with the histological examination, confirming great protection against inflammatory damages induced by TPA.

## 4. Conclusions

This work demonstrates that it is possible to increase mometasone furoate anti-inflammatory activity by developing new vehiculation systems, thus improving the treatment of inflammatory skin diseases. In this study, six formulations have been designed, developed and characterized. In all cases, vesicles of nanometric size, monodispersed and with a highly negative Z potential, were obtained. Only nanoliposomes made with Lipoid S75 (12%), MF (1 mg/mL) and Tween 80 (0.24%) assured the loading of high quantities of MF, as the entrapment efficiency values were >82%. Selected formulations of MF glycerosomes, glyceroethosomes and glyceroethohyalurosomes showed good stability over time and high biocompatibility, as all vesicles were able to protect against the cytotoxic effect of MF in dispersion. Moreover, the loading of MF in glyceroethohyalurosomes provided the highest protection against oxidative damages. The in vivo efficacy, assessed in a mouse skin epidermal hyperplasia model confirmed that 0.1% mometasone furoate glyceroethohyalurosomes represents the best candidate in preventing/treating skin inflammatory lesions.

Among the goals of this work was to contribute to overcome the limitations of corticosteroid administration. In this context, the development of new topical delivery forms that contribute to increase the efficacy and safety of conventional mometasone furoate is a real intellectual and scientific challenge. In this sense, it must be highlighted that proposed formulations could be potential candidates as new vehiculation systems for mometasone furoate, especially glyceroethohyalurosomes, which combine the beneficial properties of lipid systems and the polymer hyaluronic acid, in preventing/treating skin inflammatory lesions. Further studies should confirm the potential use of these systems in the clinical practice.

## Figures and Tables

**Table 1:** Composition of mometasone furoate-loaded vesicles.

Composition (%, *w*/*v*)	F1	F2	F3	F4	F5	F6
Mometasone furoate	0.1	0.1	0.1	0.1	0.1	0.1
Ethanol	-	30	30	-	30	30
Glycerol	4	10	10	4	10	10
Tween 80	0.24	0.24	0.24	0.57	0.57	0.57
Vitamin E	0.2	0.2	0.2	0.2	0.2	0.2
Lipoid S75	12	12	12	18	18	18
PBS qs	100	100	-	100	100	-
Hyaluronic acid 0.1% qs	-	-	100	-	-	100

**Table 2:** Mean encapsulation efficiency, particle size, polydispersity index and Z potential of mometasone furoate-loaded vesicles.

Liposome	Encapsulation Efficiency ± SD (%)	Particle Size ± SD (nm)	Polydispersity Index ± SD	Z Potential ± SD (mV)
F1	92.56 ± 0.58	78.22 ± 0.40	0.21 ± 0.01	−33.31 ± 1.41
F2	82.64 ± 0.41	172.51 ± 2.84	0.12 ± 0.02	−27.10 ± 0.97
F3	96.85 ± 0.59	165.38 ± 2.02	0.14 ± 0.05	−32.00 ± 0.47
F4	63.46 ± 3.92	104.14 ± 1.31	0.21 ± 0.02	−30.00 ± 0.32
F5	53.59 ± 0.19	172.34 ± 1.47	0.16 ± 0.01	−37.32 ± 0.75
F6	63.15 ± 0.15	178.07 ± 1.65	0.14 ± 0.02	−34.61 ± 0.72

(F1 and F4) are glycerosomes; (F2 and F5) and glyceroethosomes; (F3 and F6) are glyceroethohyalurosomes. Data are expressed as the mean values ± standard deviations (*n* = 6).

## Data Availability

Not applicable.
